# Obtaining Salt Stress-Tolerant Eggplant Somaclonal Variants from In Vitro Selection

**DOI:** 10.3390/plants10112539

**Published:** 2021-11-22

**Authors:** Sami Hannachi, Stefaan Werbrouck, Insaf Bahrini, Abdelmuhsin Abdelgadir, Hira Affan Siddiqui, Marie Christine Van Labeke

**Affiliations:** 1 Department of Biology, College of Science, University of Hail, P.O. Box 2440, Hail 81451, Saudi Arabia; insafafa@yahoo.fr (I.B.); abdelmuhsin@yahoo.com (A.A.); ha.siddiqui@uoh.edu.sa (H.A.S.); 2 Department of Plants and Crops, Faculty of Bioscience Engineering, Ghent University, Coupure Links 653, 9000 Ghent, Belgium; Stefaan.werbrouck@ugent.be (S.W.); MarieChristine.VanLabeke@UGent.be (M.C.V.L.)

**Keywords:** ascorbic acid, callus tissue, lipid peroxidation, antioxidant enzymes, NaCl tolerance, somaclonal variation, salt stress

## Abstract

An efficient regeneration protocol was applied to regenerate shoots on salt stress-tolerant calli lines of aubergine (*Solanum melongena*). These NaCl-tolerant cell lines were obtained by two different methods. On the one hand, the developed callus tissue was transferred to a medium with a continuous salt content of 40, 80, 120, or 160 mM NaCl. On the other hand, the callus tissue was subjected to a stepwise increasing salinity to 160 mM NaCl every 30 days. With the second method, calli which could be selected were characterized by compact growth, a greenish color, and absence of necrotic zones. When grown on salt-free medium again, NaCl-tolerant calli showed a decline in relative growth rate and water content in comparison to the control line. This was more obvious in the 120 mM NaCl-tolerant callus. Lipid peroxidase activity increased in 40 and 80 mM NaCl-tolerant calli; yet did not increase further in 120 mM-tolerant callus. An increase in ascorbic acid content was observed in 80 and 120 mM NaCl-tolerant calli compared to the 40 mM NaCl-tolerant lines, in which ascorbic acid content was twice that of the control. All NaCl-tolerant lines showed significantly higher superoxide dismutase (SOD) (208–305–370 µmol min^−1^ mg^−1^ FW) and catalase (CAT) (136–211–238 µmol min^−1^ mg^−1^ FW) activities compared to control plants (231 and 126 µmol min^−1^ mg^−1^ FW). Plants were regenerated on the calli lines that could tolerate up to 120 mM NaCl. From the 32 plants tested in vitro, ten plants with a higher number of leaves and root length could be selected for further evaluation in the field. Their high salt tolerance was evident by their more elevated fresh and dry weight, their more increased relative water content, and a higher number and weight of fruits compared to the wild-type parental control. The presented work shows that somaclonal variation can be efficiently used to develop salt-tolerant mutants.

## 1. Introduction

High salinity affects more than 1000 million hectares of land around the world, and it is one of the biggest challenges for agriculture today [1,2,3]. Excess of salt interferes with various plant physiological and biochemical processes, resulting in problems such as ion imbalance, mineral deficiency, osmotic stress, ion toxicity, and oxidative stress [4], thus reducing yield [5]. Salt stress induces oxidative stress by increasing the formation of reactive oxygen species (ROS) [6]. Plants with high levels of antioxidants have been reported to have greater resistance to the salt-induced oxidative damage [6].

Earlier investigations on tolerance or susceptibility behavior, under salinity conditions, and overall plant scale were unable to discriminate the systemic from cellular salinity tolerance process [7].

Recently, tissue culture approach has occurred as an effective method to underline the cellular mechanisms contributing to salt tolerance response through the use of in vitro obtained NaCl-tolerant lines [8].

Cell lines showing higher tolerance to salt stress have been detected in several crops species, and many biochemical mechanisms were involved in salt cells adaptation [9,10,11,12,13]. Moreover, the adoption of the tissue culture as a new useful tool in selecting salt-tolerant cell lines has contributed effectively towards the regeneration of salt-tolerant plants [9,14,15,16,17,18]. However, tissue culture is coupled with somaclonal variation, which is a genetic variation that has been generated during in vitro culture [19,20,21]. Somaclonal variation has provided a natural source for several desirable unintended impacts in in vitro cultured plants [22] and has also succeeded in selecting novel cultivars in many propagated crops [14,23,24].

Somaclonal variants general1y possess one or a few altered trait/s in the parental background. This variation was found to be widespread [25]; heritable on many occasions in diverse crops and ostensibly used in crop improvement, as the problem of havoc genetic rearrangements encountered in recombination breeding becomes minimized [26].

Previously, several factors have been assessed as potential causes of somaclonal variation, which include drought stress, nutritional deficit, and osmotic stress [27].

Eggplant (*Solanum melongena* L.) is a vegetable that is very popular worldwide. China, India, Egypt, and Turkey produced around 50 million tons in 2019 [28]. Eggplant contains phytonutrients with an interesting antioxidant, antimicrobial, and antiviral role [29]. Given that it is quite sensitive to salinity in the soil [30], efforts to make it more salt-tolerant are very important to increase its productivity. In a number of crops, in vitro selection proved to be a promising method to obtain salt-tolerant genotypes. The method is based on spontaneous or induced somaclonal variation of in vitro cultured plant cells during callogenesis. Then, a critically high salt shock or a stepwise-increased salt concentration is applied to select salt-tolerant cell lines, which are then regenerated into shoots [31,32,33,34].

In eggplant, successful somatic embryogenesis was reported [35,36,37,38] as well as shoot organogenesis [39,40,41], and it was found that high frequency and rapid regeneration protocol was developed from eggplant cotyledonary leaf. However, regeneration capacity is highly dependent on genotype [42].

As far as we are concerned with this matter, no attempts have yet been made to exploit the production and selection of NaCl-tolerant aubergine cells and regenerate plants from them. This study was conducted to regenerate cell lines tolerant to high concentrations of NaCl, assess the effective salt tolerance of the plants regenerated from them, and evaluate the potential of somaclonal variation for development of salinity-tolerant cultivar, as this is what it ultimately amounts to.

## 2. Results

### 2.1. Establishment of In Vitro Salt-Tolerant Lines

**Figure 1:**
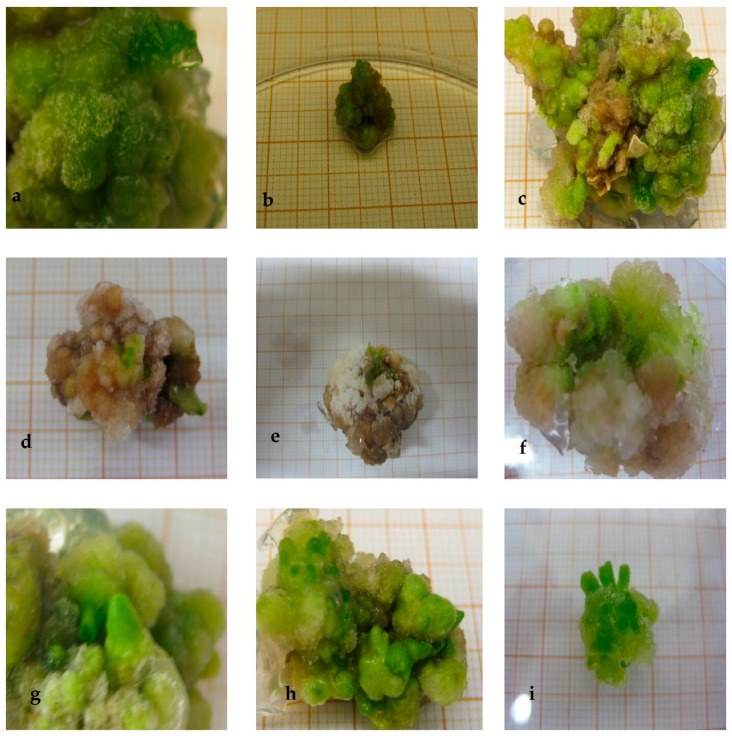
NaCl-tolerant eggplant cell lines selection obtained from unprogressive and progressive subjection to increased salinity level. (**a**) Callus tissue cultured in MS medium used for tolerant cell lines selection in absence of NaCl. (**b**) Callus tissue issued from direct subjection to 40 mM NaCl medium during 30 days of culture. (**c**) Callus tissue originated from direct exposition to 80 mM NaCl medium (presence of few necrotic parts). (**d**,**e**) Callus tissue derived from direct imposition of 120 mM NaCl salt concentration for 30 days (presence of small greenish cell clumps). (**f**) NaCl-tolerant callus tissue generated on 160 mM NaCl medium (6 months of culture). (**g**) 120 mM NaCl-tolerant callus line (6 months of culture). (**h**) NaCl-tolerant callus tissue developed on 120 mM NaCl medium following progressive selection process, for 6 months (presence of more compactness and greenish color than in (**d**,**e**)). (**i**) NaCl-tolerant callus tissue grown on 160 mM NaCl for 6 months (salt-tolerant line more abundant and greenish than in (**f**)). (**b**–**g**) Unprogressive subjection to increased salinity level. (**h**,**i**) Progressive subjection to increased salinity level.

Although both processes allowed us to select callus lines which could tolerate up to 160 mM NaCl, the results of the progressive selection were more promising. The morphology of the callus lines subject to the two selection schemes are presented in Figure 1.

The addition of 40 mM NaCl had little effect on the expansion of the callus, and the morphology remained similar to the control group (Figure 1b). Callus tissue grown in 80 mM NaCl MS medium showed less cell expansion, with some brownish parts reflecting cell necrosis (Figure 1c). On the MS medium with 120 and 160 mM NaCl, the callus hardly grew at all. After fifteen days, it developed a brown color, although surviving green groups of cells were noticed (Figure 1d,e). When salt stress was exerted again, friable light green calli emerged (Figure 1f,g). The NaCl-tolerant calli lines from the gradual selection procedure had larger cells despite an ever-decreasing growth with increasing salinity (Figure 1h,i). This selection procedure yielded dark green and a very compact calli. Even at the highest salt concentration, the resulting callus showed no cell necrosis (Figure 1i). In both selection systems, 160 mM NaCl caused too much stress. Indeed, the callus plants ceased their growth and eventually died due to the persistent stress.

### 2.2. Shoot Induction and Acclimatization

**Figure 2:**
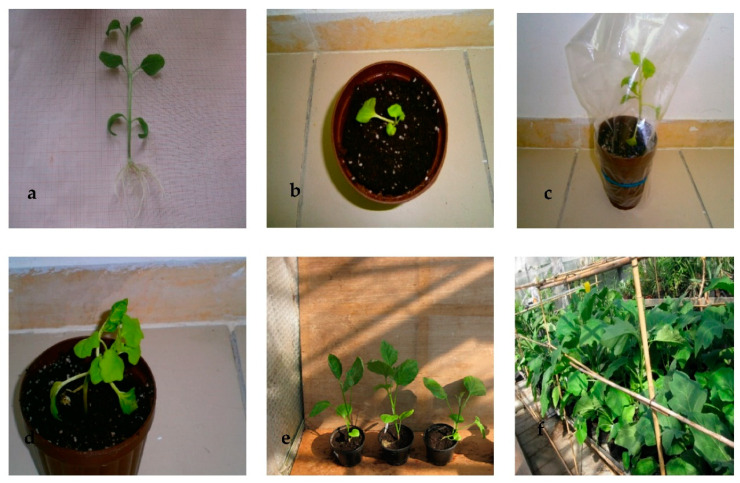
Regeneration of eggplant “Bonica F1”. (**a**) Plantlet showing a performant root system; plantlets transplanted in 2 L plastic pots filled with peat. (**b**,**c**) Freshly transferred plantlets covered with polythene plastic bags. (**d**) Hardened plantlets in the green house. (**e**,**f**) Hardened plant in the field conditions.

Shoots isolated directly from callus at 0.2 µM TDZ did not take root. They had to be precultured on a hormone-free medium before 80% efficient rooting could be achieved on MS with 0.5 µM IAA (Figure 2a). Then, plants with a normal phenotype were successfully transplanted to ex vitro conditions (Figure 2b–f).

### 2.3. Salt Stress Test

#### 2.3.1. Evaluation of Growth and Biochemical Parameters in Callus Tissue

When the 40, 80, or 120 mM NaCl-tolerant lines proliferated on the corresponding salt concentration, their growth reduction compared to the wild type line on salt-free medium was more obvious. On 40 and 80 mM NaCl, the growth was reduced by 25 and 45%, respectively. On 120 mM NaCl, the decrease in growth rate of the tolerant line was even more dramatic (75%). Compared to the control, 40 mM NaCl had no effect on the relative water content of the tolerant callus tissue. In addition, 80 or 120 mM NaCl contributed to a significant reduction in relative water content (87% and 69%) (Table 1).

A higher salt concentration always resulted in an increase of the MDA content. A salt concentration of 40 mM NaCl increased the MDA content by 13% compared to the control calli. In addition, at 80 mM NaCl and 120 mM NaCl, the MDA content increased by 145.9% and 238.8%, respectively, relative to the control. Compared to 40 mM NaCl-tolerant lines, MDA increased significantly in 80 mM and 120 mM NaCl-tolerant lines, but there was no significant difference between these two lines in terms of MDA accumulation in the corresponding lines (Table 1).

In a salt-free medium, NaCl-tolerant calli lines showed a higher significant AsA content than the control line. By increasing the salt content to 40 mM NaCl, the AsA content increased by 32% (*p* = 0.005) compared to the control line. The 80 mM NaCl medium caused the AsA content to be 2.3 times higher than the control line (*p* = 0.023). No differences in AsA content among 80 and 120 mM NaCl-tolerant calli were detected (Table 1).

Compared to the control, 40 mM NaCl, 80 mM NaCl, and 120 mM NaCl contributed to a significant increase in callus SOD enzyme activity by 21.2%, 32%, and 60.1%, respectively, and an increase in callus CAT activity by 7.9%, 67.4%, and 88.8%, respectively.

#### 2.3.2. In Vitro Salt Screening of Regenerated Plants

**Figure 3:**
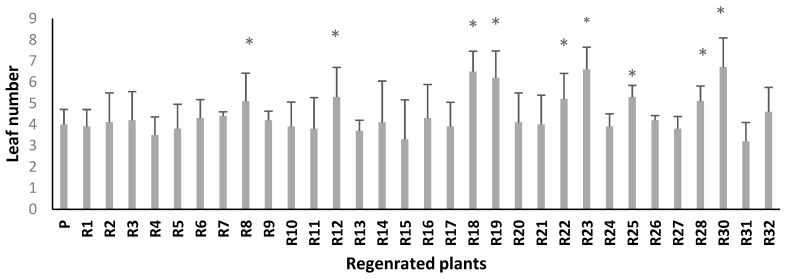
Leaf number of parent and somaclones subjected to 120 mM NaCl salt stress level. Bars represent means ± SE (*n* = 4). Significant dissimilarities between treatments (*p* = 0.05) based on Student’s *t*-test at 5% level are shown by asterisks.

**Figure 4:**
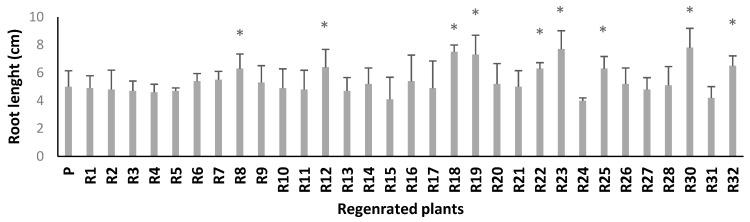
Root length of parent and somaclones subjected to 120 mM NaCl salt stress level. Bars represent means ± SE (*n* = 4). Significant dissimilarities between treatments (*p* = 0.05) based on Student’s *t*-test at 5% level are shown by asterisks.

In a medium with 120 mM NaCl, six regenerated plants (R8, R12, R18, R19, R23, and R30) had significantly more leaves than the parent line (Figure 3). In addition, the somaclonal lines R8, R12, R18, R19, R22, R23, R25, R28, R30, and R32 had significantly longer roots under the same salinity stress compared to the parent line (*p* ≤ 0.05) (Figure 4). Thus, these two criteria made it possible to select ten somaclones (R8, R12, R18, R19, R22, R23, R25, R28, R30, and R32) for further analysis.

### 2.4. Greenhouse Growth and Yield Evaluation

Concerning shoot fresh and dry weight, on salt-free medium, almost all tolerant lines performed better than the parental line. In addition, at both 80 mM NaCl and 120 mM NaCl, they were overclassing the parental line (Table 2). The plants had also longer roots than the parental control (data not shown).

The tissue water content (TWC) was significantly greater in all regenerated plants compared to the parental line in all treatments. In addition, the highest salt concentration caused TWC to be reduced significantly in parental control. However, all somaclonal lines, especially R18, R19, R23, and R30, maintained quite a stable TWC regardless of the salinity level (Table 2).

There was a significant difference in the number of fruits observed between the majority of somaclonal lines and the parental line. In the absence of salt stress, all regenerated plants except R8 produced more fruit than the parental line. Compared to the parental line, all somaclonal lines also yielded more fruit at a salt concentration up to 80 mM NaCl, at which the increase ranged from 166.6% (R32) to 333.3% (R23). At 120 mM NaCl, the fruit yield decreased in all plants, compared to 80 mM NaCl. Nevertheless, this decrease was less pronounced, especially in the regenerants R18 (8.3%), R19 (16.6%), R23 (15.3%), and R30 (25%) than in the parental line (66.6%) (Table 3). The same trend was observed with regard to the fruit weight, showing that the decline was more aggravated in the parental line (46.6%) than in the regenerants R18 (30.7%), R19 (23.5%), R23 (23.6%), and R30 (22.3%) (Table 3).

## 3. Discussion

We assume that the repeated callus culture caused somaclonal variation and that the calli lines that were developed on medium up to 120 mM NaCl had a selective advantage. Their increased tolerance went beyond physiological adaptation, as the stability of stress tolerance was further confirmed by assessing the growth of the regenerated shoots at high salinity. Similar interpretations have been made in many other in vitro salinity tolerance studies.

The progressive selection procedure seems to be more effective and adequate than the nongradual one in generating salt-tolerant eggplant calli. This might be explained by the fact that gradual subjection to increasing intensity of stress-inducing factor allows physiological and biochemical adjustments, which are the basis for a new cellular homeostasis compatible with the imposed stress. These observations are in line with those observed in rice by [15], in potato by [13], in sugarcane by [16,31], in tobacco and Medicago truncatula by [23], and in palm date by [9].

In contrast, the direct subjection to high salt stress contributed to unavoidable cellular damage. This could be attributed to irreversible cellular injuries induced by single-step exposure to a stress factor. This is an agreement with [43], who compared stepwise versus shock response to polyethylene glycol-induced low water potential in cultured potato cell-suspension cultures, Ref. [13] for potato callus cultures, Ref. [44] for chrysanthemum callus cultures, Ref. [31] for sugarcane callus cultures, and [9] in date palm callus cultures.

Salt-tolerant cell lines selections have been reported for numerous crops [9,23,45,46]. They were transferred to a regeneration medium [47] or continued to grow under salt stress to investigate the biochemical repercussions caused by the high salt content [10,33,48].

The increasing salt content reduced the growth rate of our aubergine calli lines. Such reduction in growth with high salinity could be due to induced water deficit [49] and/or toxicity associated with excessive ion uptake, particularly of Na+ and Cl−. It is well known that the growth of plants decreases under salt stress [50,51,52,53]. Growth reduction of cell tissue grown on saline medium has been reported in several previous works [9,13,31,54,55,56,57].

According to [58], the decline in growth proceeds in two specific steps: a rapid reaction to osmotic stress, and a following slow reaction induced by the influx of Na into the cellular tissue.

The tissue water content (TWC) may serve as an indicator of salt stress tolerance [52]. Increasing salinity contributes to the changing of the water balance of cellular tissue [11,59]. Increasing the salt concentration of the medium from 40 to 80 mM NaCl reduced the tissue water content of the aubergine callus. Similar responses were described by [48] in pea, by [13] in potato, by [55] in sugarcane, and by [17] in rapeseed cell lines. The reduction of the water content on a medium with increased salt content can be caused by the increase of the osmotic pressure [60,61]. Consequently, as [11,62] pointed out, the salt-tolerance level of the cellular tissue is closely linked to the callus ability to resist dehydration.

MDA is a common indicator that reflects the degree of oxidation–reduction when the plant is subjected to stress [11,52,63,64]. Lipid peroxidation is a general phenomenon that arises from exposure to salt [65]. In fact, enlarging the amount of salt contributes to increasing lipid peroxidation of the cell membrane, mainly due to the onset of oxidative stress. This might be attributed to the continuous generation of several cytotoxic reactive oxygen species (ROS) in the mitochondria, peroxisomes, and cytoplasm, which can destroy the normal cell metabolism. Many previous reports have emphasized that high free radical production (lipid peroxidation) is strongly correlated with high NaCl concentrations [10,13,21,52,66,67,68]. Plant cells have the capacity to mount a complex antioxidant defense system, both enzymatically and nonenzymatically, aiming at protecting them against salt stress [44,69,70].

Our results showed that an elevating salt concentration in the medium increased the ascorbic acid content of the resistant calli lines. This could be due to the role of AsA in protecting callus lipids and proteins against salinity-induced oxidative adversaries. This has been demonstrated in salt-tolerant cell lines from many crops [13,42,71,72]. Likewise, many salt-tolerant plants exhibit the same tendency [50,73].

Ascorbic acid (vitamin C) is an efficient antioxidant molecule and a key substrate involved in the detoxification of reactive oxygen species which helps plants adapt to stress [74,75,76,77,78,79].

In our study, increasing salinity contributed to a significant rise in the SOD activity of calli tissues, indicating the presence of a high-performance scavenging system. This is in line with several previous reports in maize [80], Acanthophyllum [16], melon [81], sugarcane [67], and Guizotia abyssinica Cass [11].

According to [80], CAT is one of the many enzymes involved in intracellular H_2_O_2_ regulation under saline conditions. In the present work, the increase in salt tolerance is related to an increasing CAT activity (Table 1), showing a high level of expression of antioxidative enzyme defense system. This is in accordance with earlier results reported by [16,31,81,82].

The in vitro screening test under 120 mM NaCl permitted the selection of ten somaclones for further evaluation in the greenhouse.

Ten regenerated plants lines (R8, R12, R18, R19, R22, R23, R25, R28, R30, and R32) showed a better salt tolerance than the parental control. This was evidenced by a higher biomass. Our findings are consistent with previous studies showing that selected somaclonal lines can stabilize the biomass of their shoots through a mechanism that can overcome salt damage at the cellular level by exclusion/avoidance [47,58,83].

Tissue water content (TWC) can be useful as a stress indicator. All somaclonal lines, especially R18, R19, R23, and R30, managed to maintain a higher and fairly stable TWC compared to the parental control, regardless of salinity. These results demonstrate that the regenerated plants are able to maintain increased turgor and cell expansion through an effective plasmolytic mechanism, thus coping with salt stress [4,47,55].

We showed that most somaclonal lines showed better yield parameters than the parental control. This was indicated by the higher number of fruits and the higher fruit weight. This could be explained by better growth or by a better nutrient distribution pattern of the somaclonal lines [9,47,67,83].

## 4. Materials and Methods

### 4.1. Initiation and Stock

Thiram-pretreated seeds of “Bonica” (Vilmorin, France) were rinsed with 70% alcohol for some seconds and washed with distilled water. Then, they were surface sterilized for 20 min in a 5% HazTab solution (1,3,5 Dichloro-Triazine-Trionedihydrate-Dichlorosodium) and 0.02% Dreft (5–15% nonionic surfactants, 15–30% anionic surfactants), followed by a solution of mercuric chloride (0.5%) for 10 min. After three rinses with sterile distilled water, the seeds were germinated on agar-solidified (0.8%) MS medium [84] with 3% (*w*/*v*) sucrose in 0.7 L glass vessels. The pH was adjusted to 5.8 with 1 N NaOH before autoclaving. Stock plants were maintained for 30 days in a growth chamber at 28 ± 2 °C and a 16 h photoperiod regime which was provided by cool-white, fluorescent lamps with a photon flux density of 36 µmol m^−2^ s^−1^.

### 4.2. Callus Induction

Leaf segments (50 mm^2^) were excised from 25- to 30-day-old seedlings. These explants were cultured for 30 days on MS medium [84] with 3% sucrose and 0.8% agar supplemented with 0.2 µM thidiazuron (TDZ). The culture was kept in a growth chamber at the same previous conditions as stock plants. Four explants per glass vessel were used, this was in four replications.

The best calli (in terms of fresh weight) formed in the presence of 0.2 µM TDZ were fragmented and then subcultured in the same fresh medium every 4 weeks for a period of 12 weeks to allow further proliferation.

### 4.3. Salt Stress Selection and Regeneration

Through two different methods, NaCl-tolerant cell lines were obtained. On the one hand, the developed callus tissue was transferred to medium with a continuous salt content of 40, 80, 120, or 160 mM NaCl. Afterwards, the callus could grow for 30 days on MS medium without salt. The obtained calli were then transferred every 30 days to MS medium with the tolerated salt content, and this was during a period of 6 months.

On the other hand, the callus tissue was subjected every 30 days to a stepwise increasing salt content in order to allow more time for a gradual selection of salt-tolerant lines. Initially, the callus was transferred to a medium with 40 mM NaCl. After 30 days, the resulting callus tissue was divided and grown in two separate media with 40 mM and 80 mM NaCl. Then, the callus tissue derived from 80 mM NaCl was subcultured in two new separate media with 80 mM and 120 mM NaCl. Finally, the callus obtained from 120 mM NaCl medium was inoculated on MS medium containing 120 mM and 160 mM NaCl. Shoots were regenerated by transferring the salt-tolerant callus to MS medium with 2 µM TDZ. Afterwards, shoots with normal morphology were cut out (1.5–2.0 cm long) and grown on hormone-free MS medium supplemented with 3% (*w*/*v*) sucrose in culture tubes. They were then transferred to MS medium supplemented with 3% (*w*/*v*) sucrose and 0.6 µM indole acetic acid (IAA) to induce root initiation. After 2 weeks of culture in a phytotron chamber, the obtained in vitro rooted shoots were moved to a greenhouse.

### 4.4. Acclimatization

The plants were transplanted in 2 L plastic pots filled with peat and covered with transparent polyethylene plastic bags for 7 days. After the emergence of new leaves, the polyethylene bags were perforated and then removed within 7 days. Next, the micropropagated plants were acclimatized after which they could grow, flower, and set seed. The fruits were weighed, and the seeds were collected by cutting the fruits under water and placing pressure on them. The extracted seeds were then washed and completely dried.

### 4.5. Salt Stress Test

#### 4.5.1. Evaluation of Growth and Biochemical Parameters in Callus Tissue

##### Growth Rate and Water Content

The growth and water content of the 40, 80, or 120 mM NaCl-tolerant lines were assessed by inoculating 1 g of each independent callus line in 380 mL glass vessels on a salt-free control medium and on a medium with the corresponding salt concentration (10 vessels per concentration, each with four lines). The growth rate of the callus was determined according to (FW_f_ − FW_i_)/FW_i_, where FW_f_ and FW_i_ were, respectively, the final and the initial fresh weight. The fresh callus was then oven dried at 80 °C for 48 h. Subsequently, the water content was calculated as (FW − DW)/FW, where DW was the dry weight. Callus tissue samples were frozen in liquid nitrogen and stored at −80 °C for further analysis.

##### Lipid Peroxidation

Lipid peroxidation was quantified as the amount of malondialdehyde (MDA) measured through the thiobarbituric acid reaction [85]. The frozen callus tissues were ground using mortar and pestle. Then, 1 g of powder was homogenized in 25 mL of 80% ethanol followed by centrifugation at 3000× *g* for 10 min. A 1 mL aliquot of sample extract was supplemented to 1 mL of thiobarbituric acid (TBA, 0.65% *w*/*v*) as well as to 1 mL of trichloroacetic acid (TCA, 20% *w*/*v*), and homogenates were incubated at 95 °C for 25 min, cooled, and centrifuged at 3000× *g* for 10 min (4 °C). The determination of the MDA amount was based on the reaction with thiobarbituric acid (TBA), and the absorption measurement was performed using a spectrophotometer (InfiniteM200 TECAN Group Ltd., Zürich, Switzerland) at λ = 440 nm, 532 nm, and 600 nm. Malondialdehyde (MDA) equivalents were calculated according to [85].

##### Ascorbic Acid Content

Extraction of ascorbic acid (AsA) was performed in 5% (*m*/*v*) metaphosphoric acid and with quartz sand at 4 °C. The homogenate was then centrifuged at 3000× *g* for 20 min at 4 °C. The concentration of AsA was determined in the supernatant according to [86]. We mixed an aliquot of 1 mL of the supernatant with 2.5 mL of 1% (*v*/*v*) freshly diluted Folin–Ciocalteu reagent. The mixture remained at room temperature for 40 min. Ascorbic acid was used as the standard for absorption at 730 nm.

##### Enzyme Assays

Fresh callus (1 g) was homogenized in 2 mL of ice-cold buffer (62.5 mM Tris–HCl, pH 6.7) containing 0.3 M sucrose using a prechilled mortar and pestle. The supernatant was centrifuged at 20,000× *g* for 30 min at 4 °C. The supernatant was used for the following enzyme activity assays.

SOD (SOD; EC 1.15.1.1) was measured by the photochemical method as described by [83]. Assays were carried out on a rotating plate under illumination. One unit of SOD activity was defined as the amount of enzyme required to cause 50% inhibition of the rate of p-nitro blue tetrazolium chloride reduction at 560 nm.

Catalase (CAT; EC 1.11.1.6) activity was quantified in a reaction mixture containing 25 mm phosphate buffer (pH 7.0), 10 mM H_2_O_2_, and enzyme. The decomposition of H_2_O_2_ was followed at 240 nm (E = 39.4 mM cm^−1^) [87].

### 4.6. Evaluation of Regenerated Plants

#### 4.6.1. In Vitro Screening Salinity Test

Thirty-two plants regenerated from calluses tolerant to 120 mM NaCl were grown for a fortnight on MS medium containing 120 mM NaCl, as well as in vitro cloned parental “Bonica F1” plants. The number of leaves and the length of the roots were recorded.

#### 4.6.2. Greenhouse Growth and Yield Evaluation

The evaluation of the impact of increasing salt stress on growth and yield parameters was carried out on 20 plants of the parental and 10 selected lines. The experiment was designed as a randomized complete block design, with four blocks. Each experimental unit contained five plants. Three salt concentrations were applied: 0 mM NaCl (control), 80 mM NaCl, and 120 mM NaCl. After 40 days of salt stress, fresh weight (FW), dry weight (DW), tissue water content (TWC), and root length were determined. The tissue water content (TWC) was calculated as (FW-DW/FW). The fruits were harvested 45 days after flowering, then counted, and immediately weighed.

### 4.7. Statistical Analysis

Data were tested by analysis of variance using SPSS Version 25 (SPSS Inc., Chicago, IL, USA) and the means were compared using Student’s two-tailed *t*-test at a level of significance of *p* ≤ 0.05. The differences of variances were checked by Fisher’s F-test. Data were presented as an average of four replications ± standard error (SE).

## 5. Conclusions

We established an in vitro protocol to regenerate plantlets from induced calli in order to set up screening tests. Obtaining salt-tolerant aubergine cell lines was more effective by using the stepwise selection process, which elucidated many mechanisms of response to stress that maintained plant tissue vitality. NaCl-tolerant cell lines can be a feasible and efficient tool for breeding and expanding our knowledge on physiological and biochemical mechanisms involved in salt tolerance.

Somaclonal variation, as a chromosomal/epigenetic rearrangement, offers an ample scope in salt tolerance crop improvement. However, phenotypic assessment based on morphological, physiological, and metabolic traits to confirm the gain of stable adaptation is indispensable. Therefore, a more fundamental perspective could be the assessment of salt tolerance stability character in progeny of selected eggplant somaclonal variants through evaluation of agronomical, physiological, and biochemical parameters at different developmental stages.

## Figures and Tables

**Table 1:** Relative growth (RGR), relative water content (RWC), malondialdehyde (MDA), ascorbic acid (AsA), superoxide dismutase (SOD), and catalase (CAT) content in control callus tissue (0 mM NaCl) and salt-tolerant calli grown on the salinity levels on which they were selected (40 mM, 80 mM, or 120 mM NaCl); RGR and RWC are expressed relative to the obtained values on salt-free medium.

NaCl (mM) on Which Line Was Selected and Tested	RGR (% of Control)	RWC (%)	MDA (nmol g^−1^ (FW))	AsA (μg g^−1^ (FW))	SOD (µmol min^−1^ mg^−1^ FW)	CAT (µmol min^−1^ mg^−1^ FW)
0	100 ± 0.0 a	99 ± 0.57 a	1.83 ± 0.35 b	102.6 ± 1.15 c	231.6 ± 7.26 c	126 ± 2.08 c
40	75 ± 0.77 b	95 ± 1.15 a	2.10 ± 0.11 b	150.03 ± 0.57 b	280.3 ± 7.79 b	136 ± 3.78 c
80	65 ± 1.15 b	87 ± 1.15 b	4.50 ± 0.57 a	228.3 ± 0.57 a	305 ± 7.63 b	211 ± 4.93 b
120	25 ± 0.57 c	69 ± 1.15 c	6.20 ± 0.88 a	260.5 ± 1.73 a	370 ± 8.66 a	238 ± 4.40 a

Values are means ± standard errors of four replicates. Significant dissimilarities between lines based on Student’s *t*-test at 5% level are shown by different lower case letters within each salinity level (*n* = 4).

**Table 2:** Effect of increasing levels of NaCl on fresh weight, dry weight, and tissue water content of parent and somaclones.

Lines	NaCl (mM)	FW (g)	DW (g)	TWC (g H_2_O/g DW)
P	0	140.9 ± 3.2 c	25.7 ± 1.2 b	0.81 ± 0.9 b
	80	80.6 ± 3.2 c	20.3 ± 4.2 b	0.74 ± 3.2 c
	120	48.3 ± 3.5 c	15.1 ± 4.5 b	0.68 ± 2.1 c
R8	0	170.7 ± 1.6 b	30.4 ± 2.6 a	0.82 ± 1.3 b
	80	150.8 ± 2.3 b	28.4 ± 3.3 a	0.81 ± 2.1 b
	120	120.3 ±1.7 b	23.2 ± 2.7 a	0.80 ± 1.6 b
R12	0	169.6 ± 1.2 b	30.1 ± 2.2 a	0.82 ± 1.1 b
	80	158. 9 ± 2.4 b	28.2 ± 1.4 a	0.82 ± 2.3 b
	120	128.3 ± 6.2 b	23.6 ± 2.2 a	0.81 ± 2.1 b
R18	0	215.5 ± 2.7 a	31 ± 0.7 a	0.85 ± 2.7 a
	80	200.4 ± 5.1 a	29.1 ± 3.1 a	0.85 ± 1.3 a
	120	165.5 ± 2.1 a	24.2 ± 1.1 a	0.85 ± 1.1 a
R19	0	216.5 ± 2.1 a	31.5 ± 1.1 a	0.85 ± 2.1 a
	80	200.3 ± 2.0 a	29.4 ± 1.0 a	0.85 ± 0.9 a
	120	166.4 ± 2.0 a	24.3 ± 3.1 a	0.85 ± 2.3 a
R22	0	170.6 ± 3.7 b	30.2 ± 3.7 a	0.82 ± 0.8 b
	80	165.3 ± 2.1 b	28.3 ± 1.1 a	0.82 ± 2.3 b
	120	125.6 ±1.2 b	23.1 ± 1.2 a	0.81 ±1.7 b
R23	0	217.5 ±1.9 a	31.5 ± 2.9 a	0.85 ±1.5 a
	80	202.3 ± 5.1 a	29.4 ± 3.1 a	0.85 ± 1.3 a
	120	165.3 ± 2.1 a	24.2 ± 1.1 a	0.85 ± 0.9 a
R25	0	167.5 ± 1.2 b	30.8 ± 2.2 a	0.81 ± 1.3 b
	80	157.2 ± 2.0 b	28.7 ± 2.0 a	0.81 ± 0.8 b
	120	130.2 ± 2.0 b	24.5 ± 2.3 a	0.81 ± 1.1 b
R28	0	165.8 ± 3.7 b	29.6 ± 2.7 a	0.82 ±2.1 b
	80	152.3 ± 2.1 b	27.5 ± 2.2 a	0.81 ± 2.1 b
	120	122.4 ± 1.2 b	22.2 ± 1.5 a	0.81 ± 1.1 b
R30	0	217.1 ± 4.2 a	33.2 ± 3.2 a	0.84 ± 2.2 a
	80	201.0 ± 3.6 a	31.1 ± 3.7 a	0.84 ± 2.5 a
	120	168.0 ± 3.9 a	26 ± 3.9 a	0.84 ± 2.9 a
R32	0	160.3 ± 2.9 b	30.4 ± 1.9 a	0.81 ± 1.1 b
	80	249.3 ± 2.5 a	28.4 ± 2.8 a	0.80 ± 3.1 b
	120	120.3 ± 0.9 b	23.2 ± 0.8 a	0.80 ± 1.8 b

Values are means ± standard errors of four replicates. Significant dissimilarities between lines based on Student’s *t*-test at 5% level are shown by different lower case letters within each salinity level (*n* = 4).

**Table 3:** Yield (fruit number per plant and mean fresh weight per fruit) of parent and somaclones subjected to increasing salt stress level for five weeks and harvested 45 days after flowering.

Parameter	NaCl (mM)	P	R8	R12	R18	R19	R22	R23	R25	R28	R30	R32
Fruit number	0	6 ± 1.8 b	6 ± 1.3 b	7 ± 1.4 b	10 ± 0.5 a	10 ± 1.2 a	6 ± 1.2 b	12 ± 1.9 a	6 ± 1.2 b	8 ± 1.3 b	10 ± 1.4 a	7 ± 0.5 b
	80	3 ± 1.6 c	8 ± 1.3 b	10 ± 1.1 b	12 ± 1.1 a	12 ± 1.5 a	8 ± 3.2 b	13 ± 2.6 a	8 ± 2.2 b	9 ± 1.4 b	12 ± 1.3 a	8 ± 0.9 b
	120	1 ± 1.1 c	7 ± 1.2 b	8 ± 1.1 b	11 ± 2.3 a	10 ± 1.7 a	7 ± 2.4 b	11 ± 2.2 a	7 ± 1.8 b	7 ± 1.3 b	9 ± 1.1 a	7 ± 1.1 b
Fruit weight	0	180 ± 1.2 b	200 ± 1.6 b	210 ± 1.1 b	250 ± 2.8 a	235 ±1.3 a	220 ±3.2 a	240 ± 2.9 a	200 ± 2.2 b	210 ± 1.1 b	220 ± 2.4 a	180 ± 2.3 b
	80	150 ± 3.6 c	210 ± 2.3 b	230 ± 2.2 b	260 ± 3.1 a	242 ±2.3 a	230 ±2.3 b	255 ± 2.1 a	205 ± 1.2 b	223 ± 1.3 b	228 ± 2.5 b	195 ± 1.8 b
	120	80 ± 0.9 c	150 ± 1.9 b	160 ± 2.9 b	180 ± 1.8 a	185 ± 2.6 a	145 ±1.9 b	195 ± 1.8 a	175 ± 2.5 a	164 ± 0.7 b	177 ± 0.9 a	143 ± 1.5 b

Values are means ± standard errors of four replicates. Significant dissimilarities between lines based on Student’s *t*-test at 5% level are shown by different lower case letters within each salinity level (*n* = 4).

## Data Availability

Not applicable.
